# Cocoa's Residual Honey: Physicochemical Characterization and Potential as a Fermentative Substrate by* Saccharomyces cerevisiae* AWRI726

**DOI:** 10.1155/2019/5698089

**Published:** 2019-02-03

**Authors:** Paula Bacelar Leite, Wanderson Mariano Machado, Alaíse Gil Guimarães, Giovani Brandão Mafra de Carvalho, Karina Teixeira Magalhães-Guedes, Janice Izabel Druzian

**Affiliations:** ^1^Department of Chemical Engineering, Polytechnic School, Federal University of Bahia, Aristides Novis Street, N° 2, Second Floor, Federação, 40210-630, Salvador, BA, Brazil; ^2^Department of Bromatological Analysis, Faculty of Pharmacy, Federal University of Bahia, Barão de Geremoabo Street, s/n, Ondina, 40171-970, Salvador, BA, Brazil; ^3^Department of Technology, State University of Feira de Santana, Transnordestina Avenue, New Horizon, 44036-900, Feira de Santana, BA, Brazil

## Abstract

This study aims to characterize the physicochemical properties of cocoa's residual honey and evaluate its fermentative capacity as a substrate, using* Saccharomyces cerevisiae* AWRI726 as the starter culture for alcoholic fermentation. The research hypothesis was that cocoa's residual honey can be used for the production of fermented beverages. Cocoa's honey has 14.14 g.100 mL^−1^ of dry material, containing 11.80 g.100 mL^−1^ of carbohydrates and 1.20% crude protein, in addition to other minor components, such as pectin, lipids, and Fe, Mn, Na, and Zn, with a carbon-to-nitrogen (C/N) ratio (9.8) most suitable for fermentation. Fermentation at 20°C for 240 hours produced a liquid with 16% v/v ethanol (14 g.L^−1^ in 144 h). However, 24 hours of fermentation produced the maximum ethanol yield (0.373 g.g^−1^) and volumetric productivity (0.168 g.L^−1^.h^−1^), which were associated with a significant increase in the specific cell growth rate.* Saccharomyces cerevisiae *AWR1726 performed satisfactorily in the alcoholic fermentation of cocoa's residual honey, similar to that observed in other fruit beverages, thus suggesting the suitability of cocoa's residual honey for future commercial applications.

## 1. Introduction

*Theobroma cacao* L. (Sterculiaceae) is an economically important crop in several tropical countries. In Brazil, the cocoa agroindustry occupies an area of 672.435 ha and generates 52.413 tons of residue per year [1]. Cocoa cultivation has always been associated with the production and economic exploitation of its almonds for chocolate production [2–4]. Chocolate processing creates a significant amount of waste, primarily during fruit breakage and in the extraction of liquid from the pulp, which is known as cocoa's residual honey. Cocoa's residual honey is an opaque yellow mucilaginous liquid that is separated from the pulp enveloping the cocoa grains by simple extraction before fermentation commences. This liquid has a sweet-sour flavor; a high content of reducing sugar; and a significant amount of dietary fiber, flavonoids, and vitamin C and can be considered a natural source of bioactive phenolic compounds with considerable antioxidant activity [5]. The development of new methods for processing this waste and innovative uses for this byproduct is necessary to minimize production losses, generate more profits, and promote the sustainable use of biomes.

One good application for fruit wastes is in the production of fermented foods.* Saccharomyces cerevisiae* is the principal yeast used in fermentation processes, including winemaking, breadmaking, and brewing [6–8]. A variety of agroindustrial waste biomasses have been used as raw materials for fermentation, including cashew apple fruit [9], soursop [10], bananas, oranges, cherries, and mangoes [11].

Thus, this study aims to characterize the physical and chemical properties of cocoa's residual honey and to evaluate its fermentative capacity as a substrate, using* S. cerevisiae* AWRI726 as the starter culture for alcoholic fermentation.

## 2. Materials and Methods

**Figure 1:**
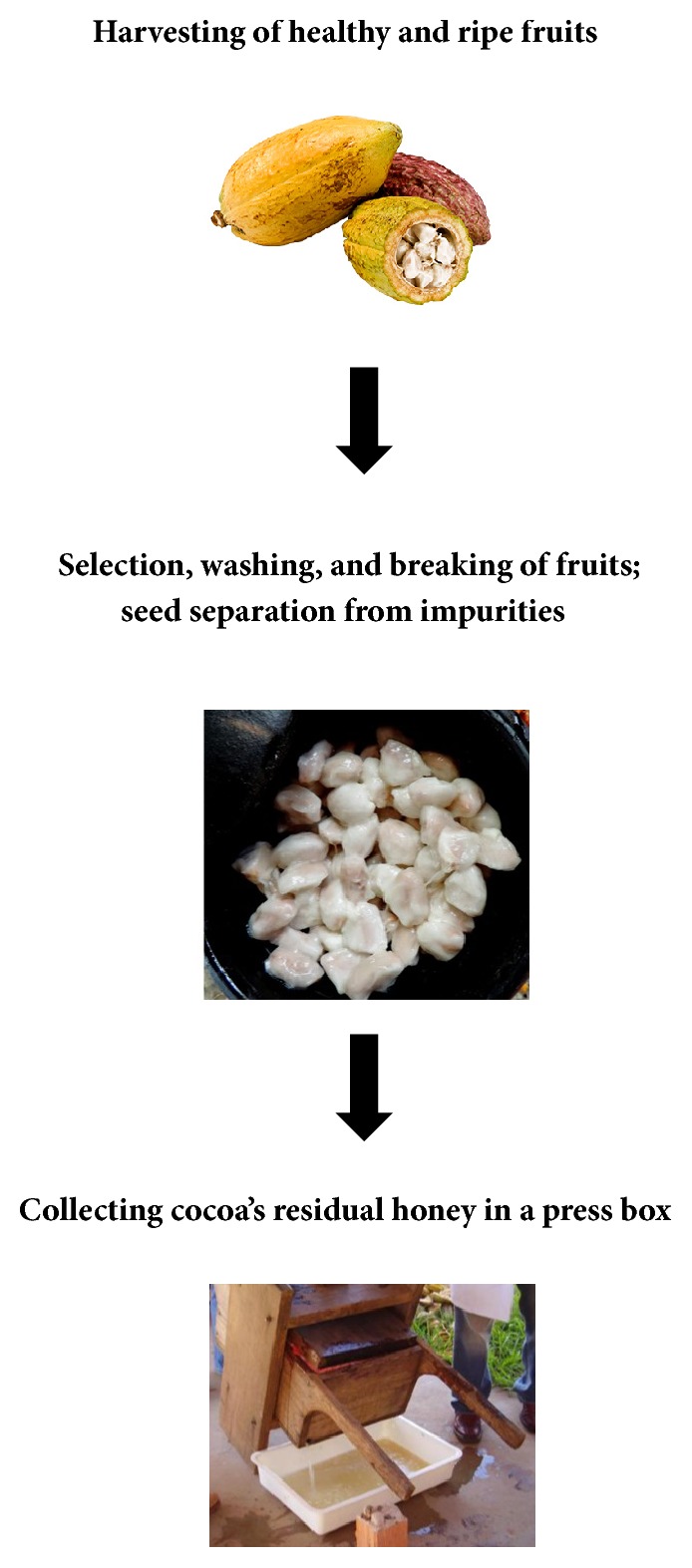
Stages for obtaining cocoa's residual honey.

The raw material of the cocoa's residual honey was donated by producers from farms in the rural area of Uruçuca, Bahia, Brazil (latitude: 14° 47′ 20′′ S; longitude: 39° 02′ 58′′ W; altitude: 52 m). The cocoa honey was collected before cacao bean fermentation (Figure 1), pasteurized at 70°C for 20 min, and frozen at −20°C. The dry commercial yeast* S. cerevisiae* AWRI726 was obtained from the bakery industry (Maurivin City Brand, Australia) and was used for the controlled fermentation of cocoa's residual honey.

### 2.1. Physicochemical Characterization of Cocoa's Residual Honey

The determination of soluble solids, expressed as °Brix, was performed in a portable digital refractometer (Biobrix, MecLab). The pH was determined with a benchtop pH meter (Hanna Instruments, HI 221). The moisture, ash, total lipids, protein, acidity, and calcium pectate (gravimetric method) were determined according to AOAC methods [16].

### 2.2. Mineral Profile of Cocoa's Residual Honey

The mineral profile of cocoa's residual honey was determined according to the method used by Brito et al. [17]. Levels of the elements Ba, Fe, Mn, Al, Cu, Na, Sr, Zn, K, Ca, Mg, and P were measured using Inductively Coupled Plasma Mass Spectrometry (ICP-MS) using an XSeries 2 ICP-MS (Thermo Fisher Scientific, Inc.).

### 2.3. Preparation of Cocoa's Residual Honey Must

The methodology proposed by Dias et al. [13] was used for must preparation. Cocoa's residual honey was thawed at room temperature and added to a sucrose solution to adjust the sugar concentration to 19 °Brix. Calcium carbonate (CaCO_3_) was added to increase the pH value to 5.0. Sulfur dioxide in the form of potassium metabisulfite was added up to a concentration of 200 mg.L^−1^ free SO_2_ to inhibit bacterial growth.

### 2.4. Submerged Fermentation of Cocoa's Residual Honey by S. cerevisiae AWRI726

*S. cerevisiae* AWRI726, a dry commercial yeast strain, was used in the fermentative process. The yeast was reactivated by precultivation in YEPD medium (1% yeast extract, 2% peptone, and 2% glucose) for 24 h at 28°C. After 24 h of incubation, the medium was centrifuged at 150 rpm and the biomass was used to inoculate 250 mL of YEPD at a concentration of 1.0 × 10^6^ g.L^−1^. The concentration was determined from cell counts obtained using the Neubauer chamber technique, while cell viability was assessed using methylene blue staining according to Dias et al. [13]. Fermentation of 2.250 mL of cocoa's residual honey was conducted at 20°C for 240 hours, in triplicate. The refractive indices of sucrose, glucose, fructose, and ethanol were monitored every 24 hours by high-performance liquid chromatography-refractive index (HPLC-RI) (Perkin-Elmer Series 200) in fermentation cultures. Samples of 10 mL were collected every 24 hours.

To calculate concentration of cells, 10 mL samples of the cell suspension were collected every 24 hours. The cell suspensions were centrifuged at 4000 x g for 20 minutes at 20°C. The cell pellet was recovered and washed with distilled water, centrifuged at 4000 x g for 10 minutes, and dried at 105°C to constant weight. The concentration of cells in suspension was determined by dry weight of yeast (g.L^−1^).

### 2.5. Identification and Quantification of Sugar and Ethanol Levels in the Must from Fermented Cocoa's Residual Honey

The identification and monitoring of sugars and ethanol in the fermentation process of cocoa's residual honey was conducted by HPLC-RI (Perkin-Elmer Series 200) using a pre-Polypore Ca column (30 mm x 4.6 mm x 10 *μ*m), followed by a Polypore Ca column (220 mm x 4.6 mm x 10 *μ*m), according to Dias et al. [13].

### 2.6. Response Variables in the Fermentation Process

The response variables Y_P/S_, Q_P_, and *μ*_X_ in the fermentation process are based on substrate consumption, product formation, and cell growth. The ethanol production yield factor (Y_P/S_), ethanol volumetric productivity (Q_P_), and specific cell growth rate (*μ*_X_) were calculated using

(1) YP/S=P−P0S0−S

(2) QP=ΔPt

(3) ln⁡XX0=μX.t,

where

P_0_ and P are the concentrations (g.L^−1^) of the initial and final ethanol

X_0_ and X are the concentrations (g.L^−1^) of the initial and final cells

S_0_ and S are the concentrations (g.L^−1^) of the initial and final sucrose

t is time

### 2.7. Statistical Analysis

The data were subjected to analysis of variance (ANOVA) and Tukey's post hoc tests at a significance level of* p*<0.05, performed with Statistica software (version 7, StatSoft, Inc., Tulsa, OK, USA).

## 3. Results and Discussion

### 3.1. Physicochemical Characterization of Cocoa Honey

The physicochemical composition of cocoa's residual honey, which is summarized in Table 1 with comparisons to other fruit pulps, revealed a rich source of nutrients. The composition of cocoa's residual honey is very similar to that of other fruit pulps, especially that of soursop (Table 1). Among the micronutrients (Table 1), the presence of zinc (0.40 mg.L^−1^), copper (0.07 mg.L^−1^), and manganese (0.30 mg.L^−1^) in cocoa honey is important because of the positive effect they have on the respiratory activity and growth rate of* S. cerevisiae*. Stehlik-Tomas et al. [18] report that mineral composition has an important role in cellular metabolism, mainly due to the requirement of some minerals as cofactors for several enzymes. The total soluble solid (13.30 °Brix) in cocoa's residual honey is significantly (*p* <0.05) lower than that of cocoa pulp, which is reported to be 20.50 °Brix [13].

Approximately 85% of the dry matter of cocoa's residual honey is represented by carbohydrates (Table 1). The concentration of free sugars is a function of the cultivar and the age of the fruit, with unripe pods containing higher proportions of sucrose and ripe pods containing mainly fructose and glucose [5]. The levels of glucose and fructose quantified by HPLC (Table 1) were 4.58±0.12 g.100 mL^−1^ and 3.25±0.03 g.100 mL^−1^, respectively (y_glucose_ = 3.10^7^ x + 13277, R^2^ = 0.9902; y_fructose_ = 6.10^7^x – 12950, R^2^ = 0.9992). Sucrose was not detected (y_sucrose_ = 5.10^7^ x – 613385, R^2^ = 0.9592).

The pectin content of cocoa's residual honey was significantly lower (*p* < 0.05) than that of the cocoa pulp evaluated by Dias et al. [13]. Pectin content, other polysaccharides (1 to 2% [19]), and the consequent viscosity of cocoa's residual honey can limit air diffusion during fermentation. The quantification of pectin in alcoholic beverages from fruits is important because of the action of pectinesterase enzymes, which release methanol. Methanol inhibits the growth of* S. cerevisiae*, even at low concentrations [13]. In addition, methanol is toxic to humans [13]. Many countries have enacted legislation to limit the methanol content in alcoholic beverages. The methanol limit depends on the type of beverage; for example, the limit of vodka is 10 g/hL, the limit of grape marc spirit/grappa is 1000 g/hL p.a., and the limit of fruit spirits, dependent on the type of fruit, is 1000–1500 g/hL p.a. [20].

The C/N ratio of 9.83 obtained for cocoa's residual honey (Table 1) is considered the most suitable for fermentation. Malt extract, which is rich in maltose, has a C/N ratio of 28.15, and peptone, a known protein source for fermentation, has a C/N ratio of 3.15 [21]. Castro et al. [22] demonstrated that C/N ratios above 10 are beneficial for amylase, protease, cellulase, and xylanase actions. On this basis, the comparison of results shown in Table 1 reveals that the present C/N ratio of cocoa's residual honey is in the range of a good substrate for fermentation. However, if the goal is to produce beverages with a high alcohol content, supplementation with sugar would be needed. A range of alcohol levels can be achieved with the supplementation of different sugar concentrations.

The pH of the cocoa's residual honey (3.51, Table 1) was similar to the values obtained in juice from the mucilage of cocoa beans from farms around Abidjan in the southern region of Ivory Coast (3.75±0.81 [12]). Compared to cocoa pulp (pH = 3.20), the pH value of cocoa's residual honey is significantly different at the 5% level [13], but it does not differ significantly from the pulp of other fruits such as soursop at pH 3.53 [14]. The pH of the cocoa's residual honey (3.51, Table 1) confers the beneficial effect of controlling bacterial contamination [23].

### 3.2. Submerged Fermentation of Cocoa Honey by the Commercial Yeast Strain Saccharomyces cerevisiae AWR1726

**Figure 2:**
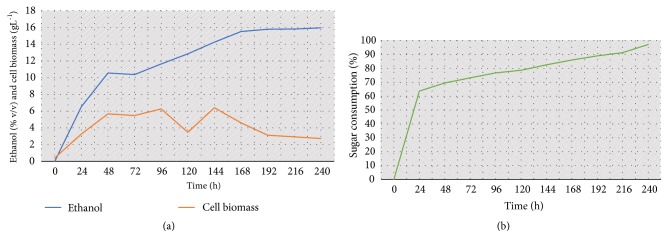
Production of ethanol and cell biomass (a) and the total percentage of sugar consumption (b) during submerged fermentation of cocoa's residual honey by* S. cerevisiae* AWRI726 (19° Brix, 20°C, and pH 5.0).

The fermentation processing of the cocoa's residual honey (19 °Brix) was conducted at 20°C for 240 hours, and the free sugar and ethanol content and cell biomass were monitored every 24 hours (Table 2 and Figure 2).

Ethanol is the major component of alcoholic beverages and determines the viscosity (body) of the wine, while also acting as a fixative [24]. Ethanol yield depends on the initial total sugar concentration in the fruit, which is measured as the total dissolved sugar concentration in the liquid must [24]. Maximum ethanol production was obtained at the end of the process at 240 hours (Figure 2(a)). However, watching the alcohol content vary between 4 and 14% v/v (20°C), the percentages established by the Brazilian legislation for fermented alcoholic beverages from fruits [25], the fermentation process of the wort from cocoa's residual honey may be interrupted for up to 144 hours for a maximum alcohol content of 14% v/v. The present work may help further studies develop the sensory analysis of the beverage and, preferably, provide an alcohol content of 10% v/v, for example, for the alcohol obtained in 48 hours. Sucrose was completely consumed by the end of the 240-hour process (Table 2). Glucose and fructose showed levels of 0.84 g.L^−1^ and 4.21 g.L^−1^, respectively, at the end of the process (Table 2). Total sugar was consumed to approximately 100% in 240 h of fermentation (Figure 2(b)).

Table 3 presents the results of the yield of ethanol production (Y_P/S_), volumetric productivity (Q_P_), and specific cell growth rate (*μ*_X_). Twenty-four hours of fermentation produced the maximum ethanol production yield (0.373 g.g^−1^) and volumetric productivity (0.168 g.L^−1^.h^−1^), with a significant increase in specific cell growth rate, which is also the phase of higher substrate consumption. Between 24 and 48 hours, yeast cells were in the exponential phase of growth, growing at a high rate, and cells had adapted to the medium (Table 3). Agroindustry biomasses have been used as raw materials for a variety of bioprocesses via fermentation by* S. cerevisiae*, and they present great potential for the production of beverages [9–11, 15]. An additional advantage of beverages developed with cocoa's residual honey is the possibility of retaining high levels of total flavonoids (7.19±0.01 *μ*g.mL^–1^), vitamin C (10.90±0.15 mg.100 mL^–1^), and antioxidant activity (EC50 DPPH= 64.74 *μ*g.mL^–1^), as related by Silva et al. [5].

The results of the fermentation of cocoa's residual honey confirmed that* S. cerevisiae* is capable of producing the minimum amounts of ethanol required (approximately 14% vol.), in accordance with usual winemaking practices in wineries with ranges of ~3 to 16% vol., which can be considered adequate for a wide variety of alcoholic beverages [12, 15].

## 4. Conclusions

Cocoa's residual honey presents adequate nutritional composition for use as a fermentative substrate by* S. cerevisiae* AWRI726. Sugar changes during cocoa honey fermentation followed classic fermentation kinetics, showing that the ethanol yield (YP/S) and volumetric productivity (QP) were maximized at 24 hours of fermentation. The utilization of cocoa's residual honey may constitute an interesting source of income for cocoa-producing countries. This perspective is important because cocoa's residual honey is presently a byproduct of the cocoa industry that has a strong economic potential.

## Figures and Tables

**Table 1:** Physicochemical characterization of cocoa's residual honey compared to other fruit pulps (the mean ± standard deviation).

Parameters	Cocoa's residual honey	Cocoa pulp^1^	Cocoa pulp^2^	Graviola pulp^3^	Cupuaçu pulp^4^
Total soluble solids (°Brix)	13.30±0.01^c^	16.17±0.74^b^	20.50±0.15^d^	13.98±0.54^c^	9.00±0.00^a^

Moisture (g.100 mL^−1^)	85.86±0.09^a^	85.30±8.60^a^	-* *-	80.57±0.45^b^	89.20±0.3^c^

Dry material (g.100 mL^−1^)	14.14±0.09^a^	14.70±8.60^a^	-* *-	19.43±0.45^b^	10.80±0.3^c^

Ash (g.100 mL^−1^)	0.59±0.15^a^	3.76±0.84^b^	-* *-	0.71±0.00^a^	-* *-

Total lipids (g.100 mL^−1^)	0.19±0.08^a^	3.54±0.20^b^	-* *-	0.15±0.01^a^	0.30±0.00^c^

Crude protein (g.100 mL^−1^)	1.20±0.49^a^	7.20±0.21^b^	-* *-	0.88±0.00^a^	-* *-

Carbohydrate (g.100 mL^−1^)	11.80±0.09^a^	-* *-	-* *-	17.69±0.09^b^	-* *-

Glucose (g.100 mL^−1^)	4.58 ± 0.12	-* *-	-* *-	-* *-	-* *-

Fructose (g.100 mL^−1^)	3.25 ± 0.03	-* *-	-* *-	-* *-	-* *-

Pectin (g.100 g^−1^ calcium pectate)	0.36±0.02^a^	-* *-	0.57±0.00^b^	-* *-	-* *-

pH	3.51±0.04^a^	3.75±0.81^a^	3.20±0.02^b^	3.53±0.00^a^	3.50±0.02^a^

Titratable acidity (meq NaOH.100 g^−1^)	10.34±0.01^a^	17.0±6.28^b^	1.00±0.01^c^	1.27±0.06^d^	3.50±0.00^e^

C/N ratio	9.83	-* *-	-* *-	20.10	-* *-

Ba (mg.100 mL^−1^)	0.38 ± 0.01	-* *-	-* *-	-* *-	-* *-

Fe (mg.100 mL^−1^)	1.35 ± 0.02	-* *-	-* *-	-* *-	-* *-

Mn (mg.100 mL^−1^)	0.30 ± 0.01	-* *-	-* *-	-* *-	-* *-

Al (mg.100 mL^−1^)	0.15 ± 0.07	-* *-	-* *-	-* *-	-* *-

Cu (mg.100 mL^−1^)	0.07 ± 0.00	-* *-	-* *-	-* *-	-* *-

Na (mg.100 mL^−1^)	0.48 ± 0.04	-* *-	-* *-	-* *-	-* *-

Se (mg.100 mL^−1^)	0.19 ± 0.00	-* *-	-* *-	-* *-	-* *-

Zn (mg.100 mL^−1^)	0.40 ± 0.01	-* *-	-* *-	-* *-	-* *-

K, Ca, Mg, and P	n.d.	-* *-	-* *-	-* *-	-* *-

Average values marked with the same letter in the same line do not differ significantly (*p*<0.05) according to Tukey's post hoc test; n.d. = not detected.

^1^Anvoh et al. [12], ^2^Dias et al. [13], ^3^Oliveira et al. [14], and ^4^Canuto [15].

**Table 2:** Consumption of sucrose, glucose, and fructose and the formation of ethanol during submerged fermentation of cocoa's residual honey by *S. cerevisiae* AWRI726 (19° Brix, 20°C, and pH 5.0).

Time (h)	Sucrose (g.L^−1^)	Glucose (g.L^−1^)	Fructose (g.L^−1^)	Total sugar (g.L^−1^)	Ethanol (% v/v)	Cell biomass (g.L^−1^)	Sugar consumption (%)
0	147.51±0.45	22.98±0.12	16.37±0.03	186.78±0.19	0.00±0.00	0.40±0.03	0.00
24	61.34±0.16	1.80±0.53	4.79±0.02	67.83±0.21	6.52±0.22	3.27±0.13	63.69
48	50.35±0.55	1.78±0.16	4.96±0.11	56.99±0.25	10.56±0.33	5.67±0.01	69.52
72	42.84±0.67	1.67±0.06	5.68±0.14	50.00±0.27	10.37±0.50	5.47±0.06	73.22
96	36.04±1.15	1.58±0.03	5.88±0.02	43.31±0.31	11.64±0.50	6.27±0.25	76.81
120	32.65±0.78	1.41±0.04	5.88±0.01	39.83±0.24	12.85±0.76	3.47±0.29	78.68
144	25.34±0.63	1.35±0.06	5.86±0.02	32.43±0.21	14.24±0.53	6.40±0.09	82.65
168	18.79±0.51	1.32±0.04	6.01±0.02	26.06±0.18	15.51±0.23	4.60±0.06	86.07
192	13.13±0.55	1.22±0.03	6.11±0.10	20.41±0.17	15.79±0.40	1.13±0.09	89.07
216	8.80±0.63	1.24±0.03	6.12±0.07	16.11±0.20	15.81±0.61	2.93±0.20	91.38
240	0.00±0.00	0.84±0.05	4.21±0.30	5.02±0.05	15.96±0.63	2.73±0.03	97.32

**Table 3:** Ethanol yield (Y_P/S_), volumetric productivity (Q_P_), and specific cell growth rate (*μ*_X_) during the submerged fermentation of cocoa's residual honey by *S. cerevisiae* AWRI726.

Time (h)	Y_P/S_ (g.g^−1^)	Q_P_ (g.L^−1^.h^−1^)	*μ*_X_ (h^−1^)
0	0.000	0.000	0.000
24	0.373	0.168	0.299
48	0.000	0.000	0.034
72	0.190	0.018	0.008
96	0.348	0.013	0.002
120	0.188	0.012	0.007
144	0.199	0.009	0.001
168	0.050	0.002	0.004
192	0.005	0.000	0.024
216	0.014	0.001	0.031
240	0.000	0.000	0.011

## Data Availability

No data were used to support this study.
